# Importance of urodynamic evaluation of bladder function after secondary untethering in spina bifida patients: single center experience of 30 years

**DOI:** 10.1007/s00383-022-05297-7

**Published:** 2022-12-01

**Authors:** Luise Ciesla, Joanna Schneider, Beatriz Bañuelos Marco, Matthias Schulz, Ulrich-Wilhelm Thomale, Tamara Geppert, Katharina C. Trojan, Angela M. Kaindl, Anja Lingnau

**Affiliations:** 1 Center of Chronically Sick Children, Charité–Universitätsmedizin Berlin, corporate member of Freie Universität Berlin and Humboldt-Universität zu Berlin, Augustenburger Platz 1, 13353 Berlin, Germany; 2 Department of Pediatric Neurology, Charité–Universitätsmedizin Berlin, corporate member of Freie Universität Berlin and Humboldt-Universität zu Berlin, Augustenburger Platz 1, 13353 Berlin, Germany; 3 Institute of Cell- and Neurobiology, Charité–Universitätsmedizin Berlin, orporate member of Freie Universität Berlin and Humboldt-Universität zu Berlin, Charitéplatz 1, 10117 Berlin, Germany; 4 Department of Urology, Charité–Universitätsmedizin Berlin, corporate member of Freie Universität Berlin and Humboldt-Universität zu Berlin, Augustenburger Platz 1, 13353 Berlin, Germany; 5 Department of Neurosurgery Including Pediatric Neurosurgery, Charité–Universitätsmedizin Berlin, corporate member of Freie Universität Berlin and Humboldt-Universität zu Berlin, Augustenburger Platz 1, 13353 Berlin, Germany; 6 Berlin Institute of Health at Charité–Universitätsmedizin Berlin, Charitéplatz 1, 10117 Berlin, Germany

**Keywords:** Untethering, Urodynamic, Spina bifida, Bladder function

## Abstract

**Introduction:**

A TCS after primary closure of meningomyeloceles is a known complication of the spina bifida disease. Data on the outcome after SSCU surgery is heterogeneous and lacking standardization. Thus we aimed to find a reliable system for assessment of the bladder function before and after SSCU surgery and document postoperative outcome.

**Methods:**

A retrospective study was performed on a cohort of patients with spina bifida diagnosis. In total, 130 patients underwent 182 SSCU surgeries, 56 of those met our inclusion criteria. A classification system, including two different methods, was used. The AC system used baseline pressure and detrusor over activity to define three levels of bladder dysfunction, the second method ranked the severity of bladder dysfunction by awarding points from 0 to 2 for bladder capacity, maximal detrusor pressure during autonomous contractions, leak point pressure and vesicoureteral reflux A high score is correlated with a severe bladder dysfunction.

**Results:**

Gender distribution was equally (male: *n* = 29; 51.8%; female: *n* = 27; 48.2%). The median age at SSCU was 902 years (range 0.5–22.8 years). After SSCU, the stage improved in 11 patients (19.6%), worsened in 11 (19.6%) patients and remained the same in 34 patients (60.7%) after intervention (AC score). Non-worsening was observed in a total of 45 cases (80.4%) (*p* < 0.001). MHS score (*n* = 27, 48.2%) improved, remained unchanged (*n* = 12, 21.4%), 17 patients worsened (30.4%). Non-worsening in postoperative bladder functional outcome was demonstrated in 39 cases (69.6%) over all (*p* < 0.005). Regardless of whether bladder function is categorized by AC or MHS, postoperative outcome worsened significantly when SSCU was performed due to increasing deterioration in motor function alone (*p* < 0.05). Of the 24 cases with NOD as indication, 22 (91.7%) had an unchanged (*n* = 10; 41.7%) or improved (*n* = 12; 50.0%), meaning positive neuro-orthopedic outcome, only 2 (8.3%) deteriorated (*p* < 0.001).

**Conclusion:**

Our study presents reliable evaluation systems for bladder function in spina bifida patients. Since indications for SSCU surgery differ, it is important to know the possible effects on bladder function after this surgical procedure. Even a mild impairment of bladder function has a risk to deteriorate after SSCU surgery. Particularly interesting becomes this with regard to the fact that the prevalence of TCS might become more frequent with the rising numbers of prenatal closures of meningomyeloceles.

## Introduction

Spina bifida is a congenital abnormality of the neural tube including open spinal dysraphism (defects not covered by skin, e.g., myelomeningocele) as well as occult spinal dysraphism (e.g., lipomeningocele). Neurogenic bladder dysfunction is a common complication of patients with spina bifida. A tethered cord syndrome (TCS) with associated lesions of the nerves is suspected as the cause, whereas a distinction is made between primary and secondary TCS. Primary TCS is associated with various disease patterns such as occult dysraphism, thick filum terminale or intraspinal lipoma. TCS can be also the result of primary surgical treatment of the spinal cord defect [1]. Scar tissue forms as a consequence of the surgery and when body length growth occurs, the nerve fibers could be inflexible and under tension. This can lead to ischemic damage to the nerve tissue and a secondary TCS develops [2, 3]. As a result, neurogenic bladder dysfunction with incontinence, unmanageable detrusor pressure, inadequate bladder capacity and vesicoureteral reflux may occur or pre-existing symptoms may deteriorate [4, 5]. This can also affect the renal function [6, 7]. Furthermore, motor functions, pain or sensory disturbances of the lower extremities, gait, contractures and foot deformities may be also affected [1, 5, 8]. A therapeutic option to prevent deterioration of motor function or/and to improve bladder function and specifically protect the upper urinary tract is the surgical adhesiolysis of the spinal cord, the secondary spinal cord untethering (SSCU) [9–12].

To detect the occurrence and dynamics of the mentioned urological dysfunction at an early stage, urodynamic examinations are used as a sensitive measuring instrument and are recommended in set intervals [13]. The course of neurogenic bladder dysfunction can thus be monitored precisely [12, 14].

There seems to be a consensus among authors that SSCU surgery leads to improvement in urological dysfunctions in children with TCS after surgical release [9–12, 15].

The aim of our study was to investigate whether scores can be used to create a simple tool for assessing the extent of neurogenic bladder dysfunction to make a more reliable prognosis about the possible effects of SSCU surgery. We also analyzed the influence of the indication for surgery (in terms of deterioration of bladder function and/or motor function) on postoperative bladder function.

## Materials and methods

A retrospective study was performed on a cohort of patients with spina bifida diagnosis. All patients were treated at the Center for Chronically Sick Children at the Charité University Medicine Berlin, Germany, between 1990 and 2021. In total, 130 patients underwent 182 SSCU operations, 56 of those met our inclusion criteria. The analysis included those patients who had an urodynamic examination (including both urodynamic and cystourethrography) within one year before and after the surgical intervention. To ensure that the conditions for the urological examination before and after intervention were equal, patients who required Botox injections into the bladder at least five months before intervention were excluded. Urodynamic parameters of filling cystometry such as bladder capacity, detrusor pressure, leak point pressure and compliance were obtained from the medical records. If present, vesicoureteral reflux was noted. Video urodynamic examinations were performed according to the standards recommended by the International Continence Society (ICS) [16].

The anatomic level of the spina bifida lesion was defined by the first split vertebral arch on spinal MRI scans.

We divided the cohort into four groups based on the individual indication for SSCU surgery: worsening of the bladder function (BF), deterioration of the neuro-orthopedic symptoms (NOD), both (BF + NOD) and other (e.g., preliminary to spondylodesis). Patients who had increasing bladder management problems prior to untethering were grouped as BF. Patients who suffered from progressive worsening of motor function, joint deformities, reduced walking distance, spasticity or pain formed the group of NOD. Patient assessment was performed during a medical examination by a neuro-pediatrician as part of the regular follow-up visits.

**Fig. 1:**
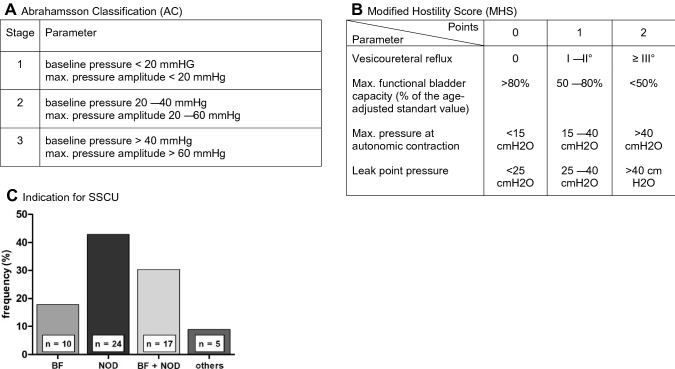
Assessment scores and indications for SSCU. **A** Abrahamsson Classification (AC); impairment of bladder function: stage 1 = mild, stage 2 = moderate, stage 3 = severe. **B** Modified Hostility Score (MHS); impairment of bladder function: 0–1 points = mild, 2–4 points = moderate, > 5 point = severe. **C** Frequency of indications for SSCU. Deterioration of: bladder function (BF), neuro-orthopedic disorders (NOD), bladder function and neuro-orthopedic disorders (BF + NOD) and others (*n* = 56)

To the bladder function before and after SSCU operations and between patient groups, a classification system, including two different methods, was used. The first method (Abrahamsson classification = AC) used baseline pressure and detrusor over activity to define three levels of bladder dysfunction, which refers to the categorization used by Abrahamsson et al. (Fig. 1A) [17]. The second method ranked the severity of bladder dysfunction referring to a modified Hostility Score (MHS) from the AWMF Guidelines [18] and an objective score by Galloway et al. [19]. The MHS awarded points from 0 to 2 for bladder capacity, maximal detrusor pressure during autonomous contractions, leak point pressure and vesicoureteral reflux (Fig. 1B). A high score is correlated with a severe bladder dysfunction.

We used SPSS Statistics (version 24) for all statistical calculations and GraphPad Prism (version 7) to design the figures. Categorical data were compared using the chi-squared test. Test results with a *p* value < 0.05 were considered statistically significant.

## Results

In our cohort 130 patients underwent 182 SSCU. Only 56 interventions in 52 patients met the inclusion criteria. Mean follow-up was 5.5 ± 3.2 months. Gender distribution was equally (male: *n* = 29; 51.8%; female: *n* = 27; 48.2%). The median age at SSCU was 9.02 years (range 0.5–22.8 years). Closed spinal dysraphism was observed in 10.7% (*n* = 6) of surgeries, a spina bifida aperta in 89.3% (*n* = 50). Most interventions were performed on patients with a lumbar lesions (*n* = 39; 69.6%), followed by sacral (*n* = 13; 23.2%) and thoracic (*n* = 4; 7.1%) lesion levels. The reasons for SSCU were: BF (*n* = 10; 17.9%), NOD (*n* = 24; 42.9%), BF + NOD (*n* = 17; 30.4%), and other indications (*n* = 5; 8.9%) (Fig. 1C).

### Benefit in bladder functional outcome in patients after SSCU

**Fig. 2:**
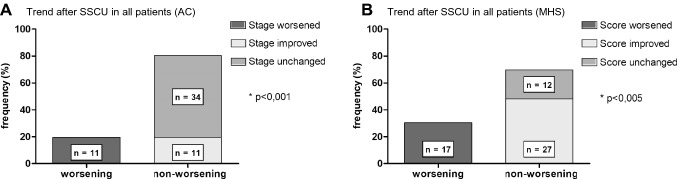
Bladder functional outcome after SSCU. **A** AC: worsening vs. non-worsening in the stage in all patients after SSCU, * *p* < 0.001, chi-squared test (*n* = 56). **B** MHS: worsening vs. non-worsening in the score in all patients after SSCU, * = *p* < 0.005, chi-squared test; (*n* = 56)

Regarding AC, 9 out of 56 procedures (16.1%) were classified as stage 1 preoperatively, 26 (46.4%) as stage 2 and 21 (37.5%) as stage 3. After SSCU, the stage improved in 11 patients (19.6%), worsened in 11 (19.6%) patients and remained the same in 34 patients (60.7%) after intervention. As we considered stable bladder function as well as improved outcome after surgery to be a success of SSCU, we defined an improved stage and an unchanged stage after surgery as non-deterioration. This condition was observed in a total of 45 cases (80.4%) (Fig. 2A, chi^2^-test, *p* < 0.001).

With regard to the MHS, 17 of 56 interventions (30,4%) were carried out with a score between 0 and 1 before surgery, 25 (44,6%) had a score of 2 to 4 and 14 interventions (25%) were performed with an initial score of 5 or more. The minimal score was 0, the maximum value was 7 points before the intervention and 6 points after. In nearly half of the cases (*n* = 27, 48,2%) the score improved, as measured by a decrease in MHS, or remained unchanged in 12 patients (21.4%), 17 patients worsened (30.4%). Non-worsening in postoperative bladder functional outcome was demonstrated in 39 cases (69,6%) over all (Fig. 2B, chi^2^-test, *p* < 0.005).

### High risk for worsening of bladder functional outcome after SSCU due to the neuro-orthopedic indication (NOD)

**Fig. 3:**
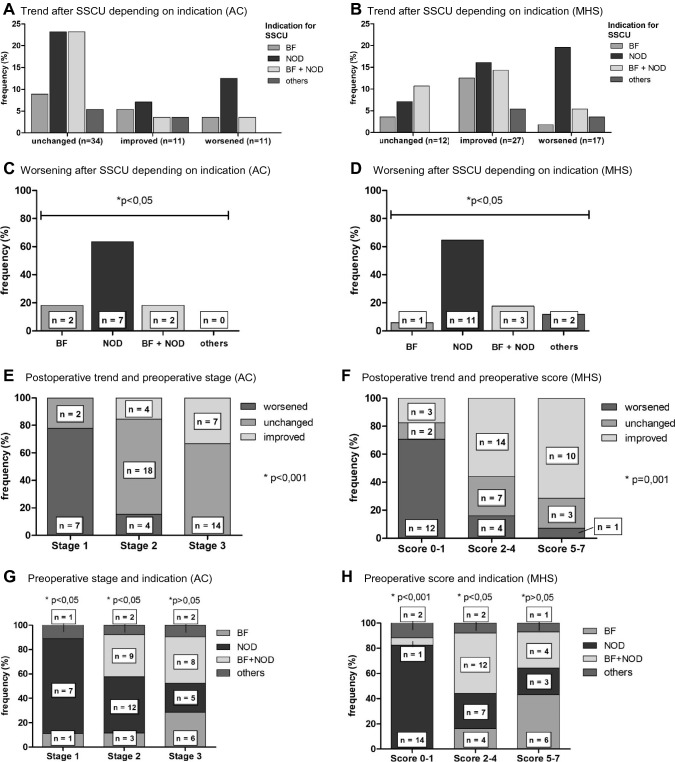
Bladder functional outcome after SSCU depending on indication and preoperative bladder situation. **A** AC: indications by postoperative stage trend. **B** MHS: indications by postoperative score trend **C** AC: Worsened bladder functional outcome after SSCU by indication, *p* < 0.05, chi-squared test. **D** MHS: worsened bladder functional outcome after SSCU by indication, *p* < 0.05, chi-squared test. NOD hold a significant majority in worsened bladder functional outcome in AC and MHS after SSCU. **E** AC: postoperative trend depending on preoperative stage, *p* < 0.001, chi-squared test. **F** MHS: postoperative trend depending on preoperative score, *p* < 0.001, chi-squared test

When considering the postoperative deterioration of bladder outcome depending on the indication for SSCU, the NOD group dominates regardless of the chosen assessment score. (Fig. 3AB). Categorized with AC, 11 times a surgery lead to a worsened bladder functional outcome. In 7 of these 11 cases (63,6%) the indication for surgery were NOD, the remainder were BF and BF + NOD, twice each (18.2% respectively) (Fig. 3C). Referring to the MHS, 17 interventions resulted in worsening, 11 of them with NOD as indication (64.7%), one with BF (5,9%), 3 with BF + NOD (17.6%) and 2 times others (11.8%) (Fig. 3D). We further analyzed those 17 patients, listed details on pre and postoperative scores, intications for surgery and postoperatibe outcome (Table 1). Regardless of whether bladder function is categorized by AC or MHS, postoperative outcome worsened significantly when SSCU was performed due to increasing deterioration in motor function alone (chi^2^-test *p* < 0.05).

**Table 1:** Details of the patients with worsening of the bladder function after untethering surgery

Patient	Indication	Score pre OP	Score post OP	Neuro-orthopedic clinical situation before surgery	Situation post OP	Positive neuro-orthopedic outcome
1	NOD	0	1	Increase in deformity of joints and loss of muscle strength in legs	Deterioration stopped	Yes
2	NOD	1	2	Increase in foot deformity and loss of muscle strength of the left leg	Deterioration stopped	Yes
3	BF + NOD	2	3	Increase in contractures and spasticity of the left leg, so that walking in the orthoses used up to that point was difficult	Activity in the right leg worse than before the operation; still walking with difficulty	No
4	Other	0	1	Due to progressive scoliosis, the use of walking orthoses was no longer possible. Goal: by tethered cord with large spinal lipoma as preparation before scoliosis surgery	No deterioration of the neuro-orthopedic clinic; in the course successful scoliosis surgery	Yes
5	NOD	0	5	Increasing back pain and pain in the scar area, increasing scoliosis	No more pain	Yes
6	NOD	2	3	Back pain after about 30 min of walking	No more pain	Yes
7	NOD	1	2	Back pain and pain in the scar area	No more pain	Yes
8	NOD	1	4	Increase in deformity of joints and loss of muscle strength in legs; myoclonia in toes in right foot	Muscle strength returned, no more myoclonia	Yes
9	NOD	1	6	Back pain and hypersensitivity with pain in the scar area, myoclonia in legs	No more pain, no more myoclonia, normal sensitivity in the scar area	Yes
10	NOD	0	1	Due to progressive scoliosis, the use of walking orthoses was no longer possible. Goal: by tethered cord as preparation before scoliosis surgery	No deterioration of the neuro-orthoaedic clinic, in the course successful scoliosis surgery	Yes
11	BF	3	6	No deterioration	No deterioration	No
12	NOD	1	2	Loss of muscle strength in legs, increasing problems with walking	Walking improved, Walking distance improved, muscle strength returned	Yes
13	BF + NOD	5	6	Loss of muscle strength in legs, increasing problems with walking	Walking improved, walking distance improved, muscle strength returned	Yes
14	NOD	0	1	Increase in deformity of joints and loss of muscle strength in legs	Muscle strength returned, walking distance improved	Yes
15	NOD	0	2	Increase in deformity of joints and loss of muscle strength in legs, increased spasticity	Muscle strength returned, walking distance improved, spasticity returned	Yes
16	Other	1	2	Due to progressive scoliosis, the use of walking orthoses was no longer possible. Goal: by tethered cord as preparation before scoliosis surgery	No deterioration of the neuro-orthopedic clinic, in the course successful scoliosis surgery	Yes
17	BF + NOD	3	5	Loss of muscle strength in legs, increased spasticity	Spasticity returned, walking improved	Yes

### SSCU surgery carried out with a good preoperative bladder situation have a significantly higher risk for a worsening of postoperative bladder functional outcome

No matter if categorized with AC or MHS we saw a similar development in the postoperative bladder functional outcome after intervention.

The preoperative bladder situation was considered good with the AC = 1 (*n* = 9 of 56; 16.1%) or a MHS of 0 to 1 (*n* = 17 of 56; 30.4%). The postoperative trend showed that 7 of 9 patients with an AC = 1 (77.8%) worsened and only 2 (22.8%) did not change (Fig. 3E, chi^2^-test, *p* < 0.001). Concerning the MHS, 12 of 17 patients with a score of 0–1 points (70.6%) deteriorated while 2 (11.8%) stayed the same and 3 (17.6%) improved (Fig. 3F, chi^2^-test, *p* < 0.01).

### Patients undergoing SSCU because of NOD benefit from surgery concerning the neuro-orthopedic outcome

After investigating bladder function in patients with NOD as an indication, we asked how SSCU affected motor function. Since the development of NOD in spina bifida patients is a dynamic and progressive process, an unchanged neuro-orthopedic status over time after surgery can be considered a success.

**Fig. 4:**
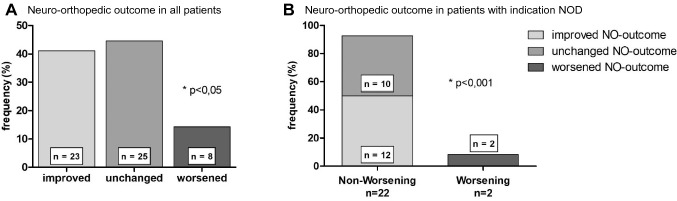
Neuro-orthopedic outcome after SSCU. **A** Frequency of postoperative trend in neuro-orthopedic (NO) outcome measured by a clinical examination; *p* < 0.05, chi-squared test; (*n* = 56). **B** Trend in postoperative neuro-orthopedic outcome in patients with the indication NOD (neuro-orthopedic disorders); *p* < 0.001, chi-squared test; (*n* = 24)

In total only 8 of 56 (14.3%) had a worsened neuro-orthopedic outcome after surgery, the majority improved (*n* = 23; 41.1%) or stayed identical (*n* = 25; 44.6%) (Fig. 4A, chi^2^test, *p* < 0.05). Of the 24 cases with NOD as indication, 22 (91.7%) had an unchanged (*n* = 10; 41.7%) or improved (*n* = 12; 50.0%), meaning positive neuro-orthopedic outcome, only 2 (8.3%) deteriorated (Fig. 4B, chi^2^-test; *p* < 0.001). Therefore, SSCU delivers a notable benefit concerning the neuro-orthopedic outcome.

## Discussion

Our data represent without exception only patients with a spina bifida diagnosis. A TCS after primary closure is a known complication of this disease. We present a reliable system for assessment of the bladder function before and after SSCU surgery. Particularly interesting gets this standardized system and tight evaluation of the bladder function in light of the fact that prenatal closure of menigomyeloceles has an increased risk of recurrent tethered cord compared to the baseline rate of tethered cord after postnatal closure [20, 21].

The effects of untethering on urodynamic findings and bladder function remain the subject of controversial discussion.

In a retrospective study Balkan et al. demonstrated that urologic disorders related to TCS can be significantly improved by a well-timed spinal cord untethering. Parameters like detrusor function (35%), electromyography recordings (45%), high leak point pressures (55%) and anal and urinary continence (70%) were improved significantly [15]. However they do not differentiate between primary and secondary TSC.

Fone et al. describe an improvement in urodynamic outcome in 75% of patients with primary TCS after surgical untethering, while in the group with secondary TCS no definite benefit was noticeable. They propose that, because in many older studies no distinction was made between the entities of TCS, this may have led to a confounding of results and a better outcome overall, since patients with primary TCS seem to benefit more from surgery [9].

In contrast to these results, Kaplan et al. presented a study with 20 patients with secondary TCS, showing an improvement in urological symptomatology in 60% [10]. Tarcan et al. found a significant increase in cystometric capacity and decrease in detrusor leak point pressure measurements in 56 children after SSCU [11]. Also, Alsowayan et al. reported significant improvement in various urodynamic parameters after SSCU [22]. Vernet et al. describe a positive trend meaning clinically stabilized or improved urodynamic status after SSCU in 64% of patients with secondary TCS. An increase in percentage of minimal acceptable total bladder capacity for age was demonstrated [12].

Houser et al. suggest a lack of improvement after surgery. They carried out cystometry before untethering and 3 to 7 days, 3 months and 6 months after intervention. While initial postoperative results in leak point pressure and compliance suggested a reduction of neurogenic bladder complaints, after 3–6 months the cystometric parameters returned to preoperative levels [23]. The study reports clinical improvement only in a small percentage of patients. However, they describe that the parameters returned to their preoperative baseline, so one could also argue that bladder function stabilized.

These examples demonstrate that since there is a heterogeneity of indications for secondary untethering, very often no clear distinction between primary and secondary spinal cord untethering and the parameters which were used to assess the urological outcomes differ, the data are difficult to compare. In our cohort e.g., we noticed that botulinum toxin A injections might also blur the urodynamic findings, therefore we excluded those patients from our analysis.

In our study, a significant majority of patients with postoperative deterioration in bladder function had only neuro-orthopedic findings (i.e., NOD) preoperatively. In addition, we saw that a good preoperative urological status was significantly more likely to deteriorate than remain stable. In about 70% of those with deteriorating bladder function, the indication for SSCU was NOD.

Knowing that tethered cord syndrome is associated with progressive worsening of symptoms, a stable, unchanged bladder condition postoperatively should be a worthwhile outcome. Therefore, we also consider an unchanged AC or MHS after intervention a success. In our cohort, a significant success, i.e., a stable or better score postoperatively, could be recorded with both of the two scales.

Our data show that postoperative worsening of bladder functional outcome is indication-dependent. This observation confirms our assumption that the preoperative status of patients, the indication for surgery and the urological scaling might be an influencing factor on the urodynamic outcome.

Nevertheless, an individual patients’ needs should always be the main consideration when deciding whether or not SSCU is beneficial. Neuro-orthopedic symptoms often lead to relevant and severe limitations of a patient’s day-to-day life. Our data suggest that, in more than 80% of patients, SSCU has a positive effect on the reduction or stabilization of these symptoms. However, since according to our data a purely neuro-orthopedic indication for untethering is associated with a higher risk of postoperative deterioration of bladder function, close urological controls should be carried out postoperatively also in this group to optimize the bladder management and adapt the therapy.

While orthopedic and neurological symptoms are usually the most noticeable ones of TCS [8, 24], the extent of neurogenic bladder dysfunction is only be detectable by specific examinations. Therefore, video urodynamic examinations are a necessary instrument to identify and monitor these dysfunctions [12, 14].

Part of our study was to investigate whether scores could be used to create a simple tool to assess the extent of neurogenic bladder dysfunction and to make a more reliable prognosis about the chances of successful SSCU.

Meyrat used a scoring system which took the parameters bladder volume, compliance, detrusor activity and vesico-sphincteric synergy into account. They demonstrated that patients with TCS had a significantly higher score than children in an age-matched control group. Eighty percent of their patients without urinary symptoms showed abnormal scores, which confirms the utility of an urodynamic scaling system to detect early neuro-urological deterioration in patients with TCS and its use in clinical practice [25]. Our MHS used similar parameters and is adding vesicoureteral reflux. The AC system takes only the baseline pressure and detrusor over activity into account [17]. Therefore we would recommend the MHS rather than the AC for urological assessments. It involves several important parameters and is more sensitive for small changes. With a single urological score, then, the development over several years can be displayed clearly. This may help noticing relevant changes and provide a basis of discussion for setting the indication for SSCU. A longer follow-up period would be desirable to better evaluate the long-term outcome of bladder function after SSCU. In our study the follow-up was limited to 5,5 ± 3,2 months postoperatively. Also patients age range was wide and the level of lesion differed.

Nevertheless, we have to consider many different parameters, the quality of life of the individual patient should have a high priority. Therefore, a well-managed and monitored neurogenic bladder dysfunction resulting from SSCU is an acceptable risk to take, if neuro-orthopedic complaints can be reduced by surgery. As of now, the best course of action is to decide on a case-by-case basis which patient can expect the maximum individual benefit from SSCU.

## Conclusion

Our study presents reliable evaluation systems for bladder function in spina bifida patients. Since indications for SSCU surgery differ, it is important to know the possible effects on bladder function after this surgical procedure. Even a mild impairment of bladder function has a risk to deteriorate after SSCU surgery. Particularly interesting becomes this with regard to the fact that the prevalence of TCS might become more frequent with the rising numbers of prenatal closures of meningomyeloceles.

## Data Availability

All data generated or analyzed during this study are included in this article. Further enquiries can be directed to the corresponding author.

## Abbreviations

SB: Spina bifida
SSCU: Secondary spinal cord untethering
BF: Worsening of the bladder function as indication for untethering
NOD: Deterioration of the neuro-orthopedic symptoms as indication for untethering
MHS: Hostility score
AC: Abrahamsson classification
